# Accounting for individual differences in human associative learning

**DOI:** 10.3389/fpsyg.2013.00588

**Published:** 2013-09-04

**Authors:** Nicola C. Byrom

**Affiliations:** Department of Experimental Psychology, University of OxfordOxford, UK

**Keywords:** individual differences, association learning, psychopathology, depression, perceptual processing, attention

## Abstract

Associative learning has provided fundamental insights to understanding psychopathology. However, psychopathology occurs along a continuum and as such, identification of disruptions in processes of associative learning associated with aspects of psychopathology illustrates a general flexibility in human associative learning. A handful of studies have looked specifically at individual differences in human associative learning, but while much work has concentrated on accounting for flexibility in learning caused by external factors, there has been limited work considering how to model the influence of dispositional factors. This review looks at the range of individual differences in human associative learning that have been explored and the attempts to account for, and model, this flexibility. To fully understand human associative learning, further research needs to attend to the causes of variation in human learning.

Research into individual differences across the human population has contributed to better understanding of everything from academic achievement to crime and delinquency, from income and poverty to health (Lubinski, 2000). Studying individual difference in human learning has contributed to our understanding of the mechanisms underlying psychopathology, particularly because learning identifies a process and therefore a mechanism by which individuals might differ. As traits of psychopathology vary across the population, our understanding of the association between psychopathology and disruptions in processes of association learning, may tell us a considerable amount about the nature and extent of variation in human associative learning. While evidence that people do not all learn the same way has been used to help us understand aspects of psychopathology, this exploration of flexibility in human learning needs to be integrated into our general understanding of the mechanisms of learning so that models can accommodate the factors that produce variance in learning. To examine individual difference in all aspects of associative learning would be too board a scope for this review. To provide focus to analysis of individual differences, this paper addresses variation in learning about combinations of stimuli. Specifically, this review presents a range of examples demonstrating individual differences in the selectivity of learning and tendency to learn about individual elements or configurations and considers how models of associative learning can accommodate this variation.

Associative learning theorists understand behavior by studying how associations between stimulus representations are acquired and used. Much of this work considers which factors influence learning and how these factors exert influence. The basic model of error prediction learning, shown in Equation (1) provides us with an indication of several factors that might influence learning. This equation was described by Rescorla and Wagner (1972).

(1) $$△V_{n}=\alpha_{n}\times \beta \times \left(\lambda -\Sigma V\right)$$

This equation describes change in associative strength of a stimulus (△*V_n_*) as a function of prediction error; that is, the discrepancy between the outcome expected following the given stimulus and the outcome that actually occurs. Prediction error is given by the difference between the asymptote of learning (λ), the total associative strength that the unconditioned stimulus (US) can support, and the current associative strength of all stimuli present on the trial. Prediction error is multiplied by the salience or intensity of that stimulus (α) and the US (β).

To provide some examples, research has considered how stimulus representations might differ on the basis of intensity and/or salience (i.e., α) and how such differences influence learning (Perkins, 1953; Logan, 1954; Redhead and Pearce, 1995). There has also been much consideration how attention shifts between different stimuli to influence learning (Mackintosh, 1975; Pearce and Hall, 1980; Le Pelley and McLaren, 2004; de Wit and Dickinson, 2009; Harris and Livesey, 2010; Lubow, 2010; McLaren et al., 2010) and how previous experiences can modify the acquisition of new stimulus representations and their associations (Kamin, 1968; Seligman, 1972; Lubow et al., 1976). This review considers whether these factors are constant across the population, or whether the influence these factors have upon learning varies between individuals. As much of the research testing individual difference in human associative learning relates to psychopathology, this review relies heavily upon illustrations from clinically focused research. The studies discussed here demonstrate substantive individual differences in central aspects of associative learning. The review concludes with a brief look at how models of associative learning can account for the observed individual differences.

## Stimulus salience and selective prediction error

Individual difference in terms of what is perceived to be salient may influence the acquisition of associations. The strength with which associative learning occurs tends to increase with stimulus salience (Kamin and Brimer, 1963; Kamin and Schaub, 1963). For instance, if two stimuli of different salience co-occur, stronger stimulus-outcome associations should be acquired for the more salient stimulus (Kamin, 1969; Mackintosh, 1971). Similarly, the strength of associative learning has been related to the strength of the unconditioned stimulus (US; Pavlov, 1927). For example, conditioned responding to shock in rabbits was observed to be directly related to the intensity of the shock, the US (Smith, 1968). To summarize with a relative simple example; a child playing with a toy may learn that pressing a lever on the toy causes a light to turn on. The perceived intensity or salience of the light (the outcome of the behavior) will influence the associative strength that can be supported. The perceived kinaesthetic experience of handling the leaver (the intensity or salience of the stimulus) will also influence the strength of learning. Variation in terms of what individuals find salient should have a substantial impact upon the acquisition of associations and may, for example, contribute to differences in associative learning in depression and anxiety.

Depression is associated with a tendency to find certain negative information salient (Matthews et al., 1995; Mogg et al., 1995; Bradley et al., 1997; Rusting, 1998, 1999; Gotlib et al., 2004; Chan et al., 2007; Phillips et al., 2010). This should have an impact upon the associations learned. Learning with salient stimuli will occur at the expense of less salient stimuli (Mackintosh, 1971). As such, if individuals with, or at risk of developing, depression find negative information more salient, they should be more likely to learn associations with negative stimuli as opposed to positive or neutral stimuli.

When learning occurs, the strength of learning that can be supported is dependent upon the strength of the outcome, or unconditioned stimulus (i.e., Rescorla and Wagner, 1972). As in the example of the child playing with a toy, the association formed between pressing the leaver and the occurrence of the outcome, the light turning on, may be influenced by how bright the light is, but also by how much lights interest the child. If the child's interest in lights is minimal, we may suggest that the perceived salience of the light, for that child, is limited. In which case, the strength of learning that the light may support should be limited. Applying this logic to individuals with depression, we may consider that the tendency to find negative information more salient may increase the perceived salience of negative outcomes. This should facilitate negative outcomes to support stronger acquisition of associative strength. This may, for instance, result in individuals with depression forming stronger associations between stimuli and negative outcomes, facilitating subsequent negative expectations. As such, the tendency to find negative information more salient may perpetuate expectation of unfavorable outcomes.

Aspects of fear conditioning associated with anxiety may be characterized by similar differences in stimulus perception. Enhanced fear conditioning is suggested to play an important role in anxiety disorders (Craske et al., 2006; Mineka and Zinbarg, 2006). Variation in the perceived intensity of a fearful stimulus is one factor that may account for differences in the ease with which fear associations are learned or maintained (Otto et al., 2007). For instance, participants' ratings of the aversiveness of a US have been observed to correlate significantly with ability to learn to dissociate a stimulus (CS) paired with the aversive US from a CS not paired with the US (Joos et al., 2013).

The salience of a stimulus, however, is not fixed. Stimulus salience may change with experience (Mackintosh, 1975; Pearce and Hall, 1980; Le Pelley and McLaren, 2004; Le Pelley et al., 2010; Pearce and Mackintosh, 2010). Learning arguably occurs more readily with stimuli that are good predictors of an outcome while stimuli that are poor predictors of an outcome lose ability to capture attention (Mackintosh, 1975). Research into mechanisms of associative learning which may underpin symptoms of schizophrenia provide examples of individual difference in changes of stimulus salience over training.

Normally, repeated presentation of a stimulus uncorrelated with an outcome retards subsequent ability to learn about that stimulus (Lubow and Moore, 1959; Lubow et al., 1976; Lubow, 2010). This effect has been termed latent inhibition. One explanation for this effect is that repeated exposure to the stimulus reduces the salience of the stimulus, specifically affecting the attentional associability of the stimulus such that the weight of attention afforded to the stimlus is reduced relative to other stimuli (Mackintosh, 1975; Le Pelley, 2004). As attentional associatibility will determine which stimulus should have access to learning and which should not (Mackintosh, 1975; Le Pelley, 2004), a reduction in attentional associability should reduce learning.

This process of latent inhibition is disrupted in schizophrenia and this disruption is associated with negative symptoms of schizophrenia in particular (Lubow et al., 1976; Baruch et al., 1988; Lubow, 1989, 2010; Gray et al., 1995; Vaitl and Lipp, 1997; Rascle et al., 2001; Gal et al., 2009). In contrast, persistent latent inhibition, that is, abnormally strong processes of latent inhibition, have been observed in animal models of positive symptoms of schizophrenia (Weiner, 2003). In contrast to the wealth of research exploring disrupted latent inhibition in human partcipants, there has been limited work exploring the effect of persistent latent inhibition in the human population. Further research would be beneficial to help understand whether mechanisms of associative learning have relevance for understanding positive symptoms of schizophrenia. The disruption of latent inhibition assocaited with negative symptoms of schizophrenia, however, suggests that negative symptoms are associated with a deficit in selective attention (Solomon et al., 1981; Weiner et al., 1981, 1984) or selective prediction error (Haselgrove and Evans, 2010).

Haselgrove and Evans (2010) have used the blocking effect to further explore the relationship between selective prediction error and schizophrenia. Blocking is thought to be dependent upon selective prediction error. Kamin (1968, 1969) observed that prior training with one stimulus interferes with the acquisition of of associative strength with a second stimulus when presented in compoud with the initial stimulus. For instance if a stimulus is paired with an outcome (**A**+) prior to pairing two stimuli with the same outcome (**AX**+), the associative strength acquired by the second stimulus (**X**) is reduced compared to a control. Selective prediction error is argued to underlie this effect (Haselgrove and Evans, 2010). The Rescorla and Wagner model of learning, described above in Equation 1, uses a summed error term and predicts that change in the associative strength of a stimulus depends upon the difference between the asymptopte of learning supported by the outcome and the associative strength of all stimuli present on a trial. For example, on the AX compound trial, A already predcits the outcome and therefore the prediction error is minmal, preventing learning with X. A failure to show blocking may suggest that prediction error is non-selective, that is, on the AX compound trial the associative strength acquired by A is not considered when learning with X and hence learning with X can occur (Haselgrove and Evans, 2010).

Blocking is disrupted in schizophrenia; this disruption is associated with the negative and depressive symptoms of schizophrenia in particular (Bender et al., 2001; Moran et al., 2008). This effect has been replicated in a non-clinical sample; individuals with high levels of introverted anhedonia, the negative symptom dimension of schizotypy, show disrupted blocking (Haselgrove and Evans, 2010). Observation of this effect with the dimension of schizotypy suggests that across the general population individuals differ considerably in the selectivity of their learning.

## Attending to the cues or the context

In an associative learning paradigm participants are usually given the opportunity to learn that a stimulus predicts an outcome. Specificity is a fundamental component of this learning. That is to say, learning that a specific stimulus, and not the context in which that stimulus is presented or any other presented stimuli, predicts that the outcome of interest. To return to the original example of a child playing with a toy; pressing the leaver causes a light to turn on. In playing with the toy the child has the opportunity to experience the contingency of leaver pressing and the occurrence of the light. Experience of this contingency should facilitate learning that a specific cue, pressing the leaver, rather than any other cue in the environment, causes the light to turn on.

One explanation for the relationship between anxiety and high levels of conditioned fear may be a deficit in specificity of learning (Baas et al., 2008; Baas, 2013). For example, if an aversive stimulus (US) is presented in a given context, it is likely that that context will be associated with that US and thus the context may begin to evoke a fear response. If the aversive US is always, and only, presented immediately after a specific cue, the cue can be used to predict the aversive US. Learning the specific association between the cue and US should reduce the association between the context and the aversive US, as the context is a less reliable predictor of the US than the cue. Failure to learn this specific association may be expected to result in continued general fear of the context. Studies have identified a relationship between learning a specific association between a threat cue and an aversive US and a reduction in general fear to the context in which the cue and aversive US are presented. Specifically, Baas (2013) observed that participants who failed to acquire an awareness of the relationship between a specific threat cue and the aversive US rated the context in which that stimulus was presented as fearful. Fear ratings for the context were reduced in participants who acquired the specific CS–US association (Baas, 2013). However, this study did not observe trait anxiety to be associated with failure to learn the specific association, though it is possible that such failure to learn the specific association may relate to characteristics of anxiety such as attentional control (Derryberry and Reed, 2002; Baas, 2013).

Individual differences in specificity of learning about cues in a context may be seen in human contingency learning. Learning contingencies allows people to make judgments about how accurately events and actions predict subsequent outcomes, allowing behavior to be guided by experience (Baker et al., 2001). While positive contingencies, where the probability of an outcome occurring increases in the presence of a stimulus, are regularly encountered, we also experience zero contingencies where the outcome is no more likely to occur in the presence than the absence of a stimulus. Accuracy in identifying zero contingencies is quite poor, especially when people are asked to consider whether their actions cause an outcome (Alloy and Abramson, 1979; Baker et al., 2010). Alloy and Abramson (1979) gave participants the opportunity to press a light switch and asked them to estimate how much control they had of a light turning on and off. There was a zero contingency relationship between pressing the light switch and the light coming on; the light was just as likely to turn on during trials where the light switch was not pressed as it was during trials where the light switch was pressed. Alloy and Abramson (1979) found that depressed participants accurately judged that they had no control of the light. Non-depressed participants incorrectly estimated that they had control of the light. This effect was termed depressive realism (Alloy and Abramson, 1979). More recent experiments exploring this effect suggest that depressed participants may be less sensitive to context information (Msetfi et al., 2005). In re-running the original Alloy and Abramson experiment, Msetfi et al. (2005) varied two factors; the outcome density and the inter-trial interval (ITI). Through this experimental design the opportunity to press a light switch and the occurrence, or non-occurrence of the light is split into trials. The ITI, that is the length of time between each trial, can be varied. Outcome density, that is the proportion of trials on which outcome occurs, can also be varied while maintaining a zero contingency. For example, in a low outcome density condition the light might turn on during 25% of the trials where the light switch is pressed and 25% of the trials where the light switch is not pressed. In a high outcome density condition the light might turn on during 75% of the trials where the light switch is pressed and 75% of the trials where the light switch is not pressed.

Varying the ITI and outcome density, Msetfi et al. (2005) observed that the original Depressive Realism effect was only present when the ITI was long and the outcome density was high. At shorter ITIs or when the outcome density was lower, non-depressed participants did not overestimate their control of the light. Interestingly, in a long ITI design participants get more exposure to the context in the absence of the outcome; that is, more experience of no-action (participants cannot press the light switch during the ITI) and no-outcome (the light never turns on during the ITI). Increasing exposure to the no-action—no-outcome contingency increases the contingency between action and outcome. As such, under these conditions, non-depressed participants were actually correct in estimating that they had control over the outcome. The failure of the depressed participants to increase their judgments of control suggests that depressed individuals were insensitive to the no-action—no-outcome information presented during the ITI (Msetfi et al., 2005; Baker et al., 2010).

## Learning about constituent elements or configurations

While linear learning refers to the acquisition and use of associations between separate stimuli and outcomes, non-linear learning refers to learning about compound stimuli as distinct configurations associated with different outcomes from those associated with the compound's constituent stimuli. The Rescorla and Wagner (1972) model of elemental learning assumes that each stimulus is processed separately so that it develops its own associative link with the outcome. When learning about, and responding to, compound stimuli, this elemental approach continues to assume that each individual stimulus develops its own associative link with the outcome. As such, the model predicts that the associative strength of a compound stimulus (i.e., Vab) is the algebraic sum of the associative strength of each of the stimuli presented (i.e., Vab = Va + Vb). While elemental theory naturally accounts for situations where the outcome following the co-occurrence of stimuli is greater than that following the separate constituent stimuli, non-linear discrimination tasks require the opposite relationship to be learnt; where the outcome following the co-occurrence of stimuli is less than, or opposite to, that following the separate constituent stimuli. Humans and animals can successfully solve non-linear discriminations, such as negative patterning (Redhead and Pearce, 1995; Shanks and Darby, 1998; Deisig et al., 2001; Myers et al., 2001; Pearce and George, 2002; Grand and Honey, 2008; Harris et al., 2008). The traditional Rescorla and Wagner (1972) instantiation of the elemental model cannot account for this. By contrast, configural theory (Pearce, 1987) can account for non-linear discrimination learning. Configural theory (Pearce, 1987) assumes that associations form between outcomes and unitary or configural representations of the pattern of stimuli present on a given trial. As such the configuration present on a compound trial (AB) should enter into an association with an outcome independent from the associative links formed between the constituent stimuli and outcomes. Though these two classes of model make contrasting predictions about how the relationship between constituent stimuli and configurations should be learnt, there is considerable support for both models, reflecting substantial variability in non-linear learning. (Melchers et al., 2008).

It has been suggested that the perceptual properties of stimuli influence whether learning will occur with separate constituent stimuli (elemental) or configurations (configural; Lachnit, 1988; Kehoe et al., 1994; Rescorla and Coldwell, 1995; Myers et al., 2001). Others have argued that these are two separate types of learning, mediated by different neural substrates (Sutherland and Rudy, 1989; Fanselow, 1999).

Several studies have looked at whether individuals differ in their tendency to learn about constituent elements or configurations. The negative patterning discrimination (A+, B+, AB−) provides a useful test of configural learning, as solving the discrimination requires participants to learn that the compound stimulus is associated with a different outcome to each of its constituent stimuli. Shanks and Darby (1998) provided a suggestion that human ability to learn non-linear discriminations, such as negative patterning, might be dependent upon rule use. Shanks and Darby (1998) demonstrated that ability to learn a negative patterning discrimination was associated with later use of rule as opposed to feature based generalization (Shanks and Darby, 1998). Rule-based generalization depends on the abstraction of and generalization from a rule. Feature-based generalization depends upon the surface similarity between separate stimuli and compounds. As such, it is assumed that rule-based generalization is more complex and might require greater understanding of the discrimination (Shanks and Darby, 1998) or more working memory capacity (Wills et al., 2011).

In the Shanks and Darby (1998) experiment participants were trained on a negative patterning discrimination (i.e., **A+**, **B+**, **AB−**) intermixed with trials where separate stimuli were paired with the outcome (i.e., **I+**, **J+**) before being asked for a prediction of the outcome following the co-occurrence of the separately trained stimuli (i.e., **IJ**?). Some participants expected the outcome to occur following the **IJ** compound, showing feature based generalization. Others demonstrated application of a negative patterning rule, expecting no outcome to occur following the **IJ** compound. Rule-based generalization was associated with strong initial discrimination learning (Shanks and Darby, 1998). Wills et al. (2011) found that individuals who completed a concurrent task while learning the same initial discrimination were more likely to show feature-based generalization (Wills et al., 2011). As such, it may be that greater working memory capacity is associated with stronger non-linear discrimination learning and rule-based generalization. Recently, Baker (2013) observed performance on the Raven's Progressive Matrices (Raven, 2000) to be associated with ability to learn a negative patterning discrimination. Ravens Matrices are designed to assess reasoning ability, and as such these results may provide support for the suggestion that rule use facilitates non-linear discrimination learning, such as negative patterning.

Negative patterning, however, essentially requires learning about a configuration (that is the co-occurrence of stimuli) independently from learning about the constituent stimuli. We may thus expect that a tendency to perceive or process groups of stimuli as a unitary configuration, and not simply a cluster of co-occurring stimuli, may influence performance. Similar task requirements have been explored in other areas of psychology. For instance, face recognition is a task thought to be reliant upon configural processing (Diamond and Carey, 1986; Tanaka and Farah, 1993; Leder and Bruce, 2000; Maurer et al., 2002). Strong face recognition has been associated with a general advantage in global processing (Macrae and Lewis, 2002; Perfect, 2003); that is, tendency to process global information prior to, or with a higher priority than, the specific elements composing the global stimuli (Navon, 1977).

As individuals differ in their tendency to show a global or local processing advantage (Navon, 1977), it is possible that such variation relates to, or influences, capacity to learn about combinations of stimuli and thus learn a non-linear discrimination. Using a similar discrimination task to that developed by Shanks and Darby (1998), Byrom and Murphy (under review) found global processing to be associated with stronger ability to learn a non-linear discrimination; specifically, individuals showing a global processing advantage were better able to discriminate **BC** from **ABC** in a modified negative patterning task (**A+**, **BC+**, **ABC−**).

## Modeling individual difference in human associative learning

Use of associative learning in exploration of clinical phenomena has advanced our understanding of mechanisms underlying cognitive aspects of psychopathology. As psychopathology is widely accepted to occur along a continuum, the clinical examples presented here contribute to the demonstration of substantial individual differences in processes of associative learning. For instance, though schizophrenia is a serious mental health problem occurring with a prevalence of around 0.4% (Saha et al., 2005; McGrath et al., 2008), schizotypy, a dimension reflecting traits of schizophrenia, varies across the population (Mason et al., 2005; Mason and Claridge, 2006). Schizotypy is, like schizophrenia, associated with disruptions in latent inhibition and blocking (Moran et al., 2003; Haselgrove and Evans, 2010) as well as impaired conditional task performance (Haddon et al., 2011) and impaired visual context processing (Uhlhaas et al., 2004; Uhlhaas and Silverstein, 2005).

Models of learning may need to account for this flexibility. If the mechanisms of associative learning vary across the population, focusing on the average performance of a sample when developing models of learning may result in models which fail to accurately capture the populations' performance. Over the years there have been many modifications to simple models of learning. While these modifications allow the models to capture a broader range of experimental findings, many different factors vary during learning and as such it may not be reasonable to search for a single modification to capture all variability in learning. It is unlikely that all factors contributing to individual differences in human associative learning could be captured by one parameter.

Individual differences in many of the factors discussed above can be captured by varying the parameters present in the Rescorla and Wagner (1972) model of learning, described in Equation (1). For instance, if individuals differ in their perception of the salience of the CS or US, modifying α or β could provide flexibility to account for this variation. Varying λ allows accommodation of individual difference in the rate of learning. Further, it may be possible to account for individual difference in selectivity of learning, as observed by Haselgrove and Evans (2010) by varying the extent to which a separable (i.e., Bush and Mosteller, 1951) as opposed to a summed (i.e., Rescorla and Wagner, 1972) error term is adopted. Variation between and integration of summed and separable error terms and the relation to processes of attention have been discussed at length elsewhere (Le Pelley, 2004; Pearce and Mackintosh, 2010).

Individual difference in ability to solve a negative patterning discrimination, however, is one example of variation that cannot be accounted for by varying existing parameters in this model. At least three different approaches have been proposed to allow for flexibility between elemental and configural models of learning; the replacement parameter, the discriminability parameter and the sampling capacity parameter. Each is discussed below.

The Replaced Elements Model (REM; Brandon et al., 2000; Wagner, 2003), conceives of stimuli as represented by multiple features or elements. The model focuses on elements that stimuli share in common and how these elements interact with elements unique to a given stimulus. In the representation of a compound there are assumed to be context independent elements which are activated whenever the stimulus is presented and context dependent elements which are activated or inhibited depending on the combinations of stimuli presented (Brandon et al., 2000). For instance, when stimulus **A** is presented alone, representations of the elements **A**_**1**_ and **A**_**2**_ may be activated. When stimulus **A** is presented in combination with stimulus **B**, the element **A**_**2**_ may be replaced by a new element, **A**_**3**_. The model adopts the stipulation that a compound should have no more capacity to elicit associative strength than any of its constituent elements. As such, in adding and inhibiting elements, the change made to the elements represented is qualitative, with the elements represented being changed, rather than a quantitative.

The replacement parameter ***r*** allows flexibility in the proportion of context dependent elements replaced when stimuli are presented in compound (Wagner, 2003). When ***r*** is 0 no replacement occurs and as such strong generalization of associative strength between stimuli and compounds is predicted. When ***r*** is 1 there is considerable replacement of elements and as such the generalization predicted to occur between compounds and constituent stimuli should be reduced. With maximal replacement of elements, the representation of the compound should be distinct from the representation of the separate stimuli.

The discriminability parameter, suggested by Kinder and Lachnit (2003) introduces flexibility into a model of configural learning (Pearce, 1987), allowing the perceived similarity between stimuli and compounds to be altered. This also affects the extent to which generalization of associative strength is predicted. The modification assumes that as it becomes harder to identify constituent stimuli within compounds, the discriminability parameter will decrease, reducing the prediction of perceived similarity between compounds and constituent stimuli (Kinder and Lachnit, 2003).

While the replacement and discriminability parameters were developed to account for the infleunce of external factors such as stimulus modality (Kehoe et al., 1994), the sampling capacity parameter was developed to account for individual difference observed in human associative learning. Sampling capacity here refers to the number of stimulus features that can be sampled on a given trial. To learn about and respond to the co-occurrence of stimuli as a distinct combination, Byrom and Murphy (under review) suggest that features of each of the co-occurring stimuli must be sampled simultaneously, such that in any given sample a configuration is represented. Variation in sampling capacity should produce variation in the extent to which the features of co-occurring stimuli can be sampled and as such result in variation in ability to represent and learn about the distinct combinations of stimuli, required to learn a non-linear discrimination. Byrom and Murphy (under review) suggest that the impact of varying sampling capacity may be modeled by incorporating a parameter, f, into a modification of Pearce's configural model of associative learning. This parameter reflects the probability of encoding a configuration, calculated from sampling capacity. For a fixed sample size, the probability of sampling a configuration of a set number of features increases as sampling capacity increases.

Pearce's (1987, 1994) configural model of learning stipulates that associative strength is acquired by the configurations of stimuli presented (i.e., **A**, **BC**, and **ABC**). However, if individuals have limited sampling capacity, they may learn about the separate stimuli and not the configurations. To allow for this flexibility, Byrom and Murphy (under review) suggest modifying Pearce's (1987, 1994) configural model of learning such that two sets of nodes may be activated by input; separate stimuli (i.e., **A**, **B**, and **C**) and presented configurations (i.e., **A**, **BC**, and **ABC**). Both sets of nodes can form associations with an unconditioned stimulus and generalization can occur between all nodes. This can be achieved by modifying Pearce's (1987, 1994) configural model of associative learning such that changes in the excitatory strength of the separate stimuli and the presented configurations is moderated by the parameter, *f*, reflecting sampling capacity. At a high sampling capacity, the excitatory strength of presented configurations changes across learning trials. At a low sampling capacity, the excitatory strength of the separate stimuli changes across learning trials. As Pearce's (1987, 1994) configural model is highly dependent on the influence of generalization, modification of this model must consider generalization, which, like change in excitatory strength, comes to be moderated by the parameter, *f*. As such, at a high sampling capacity, generalization of associative strength to separate stimuli and between presented configurations will be high, while at a low sampling capacity generalization of associative strength to separate stimuli and between presented configurations will be low, but generalization from separate stimuli to presented configurations will be high.

The extent to which parameters can be used to make predictions about learning and behavior in novel situations is dependent upon ability to specify the parameter a-priori. Each of these modifications faces challenges in specifying parameters a-priori. The replacement parameter depends on the proportion of elements replaced when a stimulus is presented in compound. The discriminability parameter depends on ability to discriminate between stimuli. It is possible that either of these parameters may be calculated for a specific stimulus set, but many factors would be expected to interact to influence “element replacement” and stimulus discriminability, limiting the extent to which these parameters can, in general, be specified a-priori. Sampling capacity may be calculated from individual difference in tendency to show local or global processing. To do this it is necessary to have relevant data, such as participants' performance on a task such as the Navon task (Navon, 1977).

## Conclusions

Individual difference in human associative learning appears to have substantial impact upon learning. To accurately understand and model human associative learning, this flexibility needs to be accounted for in terms of specific parameters. Though the introduction of new parameters to increase the flexibility of models of learning has limitations, exploring the extent to which variation in specific parameters can account for specific individual difference in human associative learning, should enhance understanding of mechanism of associative learning.

### Conflict of interest statement

The author declares that the research was conducted in the absence of any commercial or financial relationships that could be construed as a potential conflict of interest.
